# Usefulness of an intraoperative flipped monitor in laparoscopic surgery with situs inversus totalis: a case report of laparoscopic-assisted ileocecal resection

**DOI:** 10.1186/s40792-023-01806-5

**Published:** 2024-01-08

**Authors:** Ryoma Yokoi, Jesse Yu Tajima, Shigeru Kiyama, Masahiro Fukada, Ryuichi Asai, Yuta Sato, Itaru Yasufuku, Yoshihiro Tanaka, Naoki Okumura, Katsutoshi Murase, Takao Takahashi, Nobuhisa Matsuhashi

**Affiliations:** Department of Gastroenterological Surgery and Pediatric Surgery, Gifu University Graduate School of Medicine, 1-1 Yanagido, Gifu City, Gifu, 501-1194 Japan

**Keywords:** Situs inversus totalis, Laparoscopic surgery, Intraoperative flipped monitor

## Abstract

**Background:**

Situs inversus totalis (SIT) is a rare congenital condition that involves complete transposition (right to left reversal) of the visceral organs. Laparoscopic surgery can be challenging because of the mirror-image anatomy. We describe a surgical innovation in laparoscopic surgery for SIT.

**Case presentation:**

A 41-year-old man with SIT was diagnosed with an appendiceal tumor and underwent laparoscopic-assisted ileocecal resection. Preoperatively, we evaluated anatomical variations using 3D-computed tomography and simulated mirror images by watching flipped videos of patients with normal anatomy undergoing similar operations. During the operation, port placement and the surgeons’ standing positions were reversed. Additionally, two monitors were placed at the patient’s head, with one monitor showing original images, and the other showing flipped images that looked the same as the normal anatomy. We checked the range of the mobilized region and important anatomical structures by watching the flipped monitor as needed. The patient’s postoperative course was uneventful.

**Conclusions:**

Due to the complexities of laparoscopic surgery for SIT, preoperative preparation and surgical innovation are necessary for safe surgery. Several suggestions have been made to understand anatomical anomalies and improve operability; however, surgeons must focus on the mirror-image anatomy throughout the operation. Therefore, the use of intraoperative flipped monitor will be helpful for surgeons in reducing the risk of anatomical misidentification.

**Supplementary Information:**

The online version contains supplementary material available at 10.1186/s40792-023-01806-5.

## Background

Situs inversus totalis (SIT) is a rare congenital condition that involves complete transposition (right to left reversal) of the visceral organs, with a reported incidence of 1 per 5000–10000 individuals [1]. Laparoscopic surgical procedures in patients with SIT can be complicated and technically difficult due to the mirror-image anatomy and rarity, even for experienced surgeons [2, 3]. Preoperative preparation and surgical innovation are necessary for safe surgery, and several suggestions have been made to understand the unfamiliar anatomy and improve operability [2, 3]. However, surgeons must pay attention to mirror images throughout the operation because anatomical misidentification can lead to serious complications. Here, we describe a case of laparoscopic-assisted ileocecal resection of an appendiceal tumor in a patient with SIT using an intraoperative flipped monitor to reduce the risk of anatomical misidentification.

## Case presentation

**Fig. 1:**
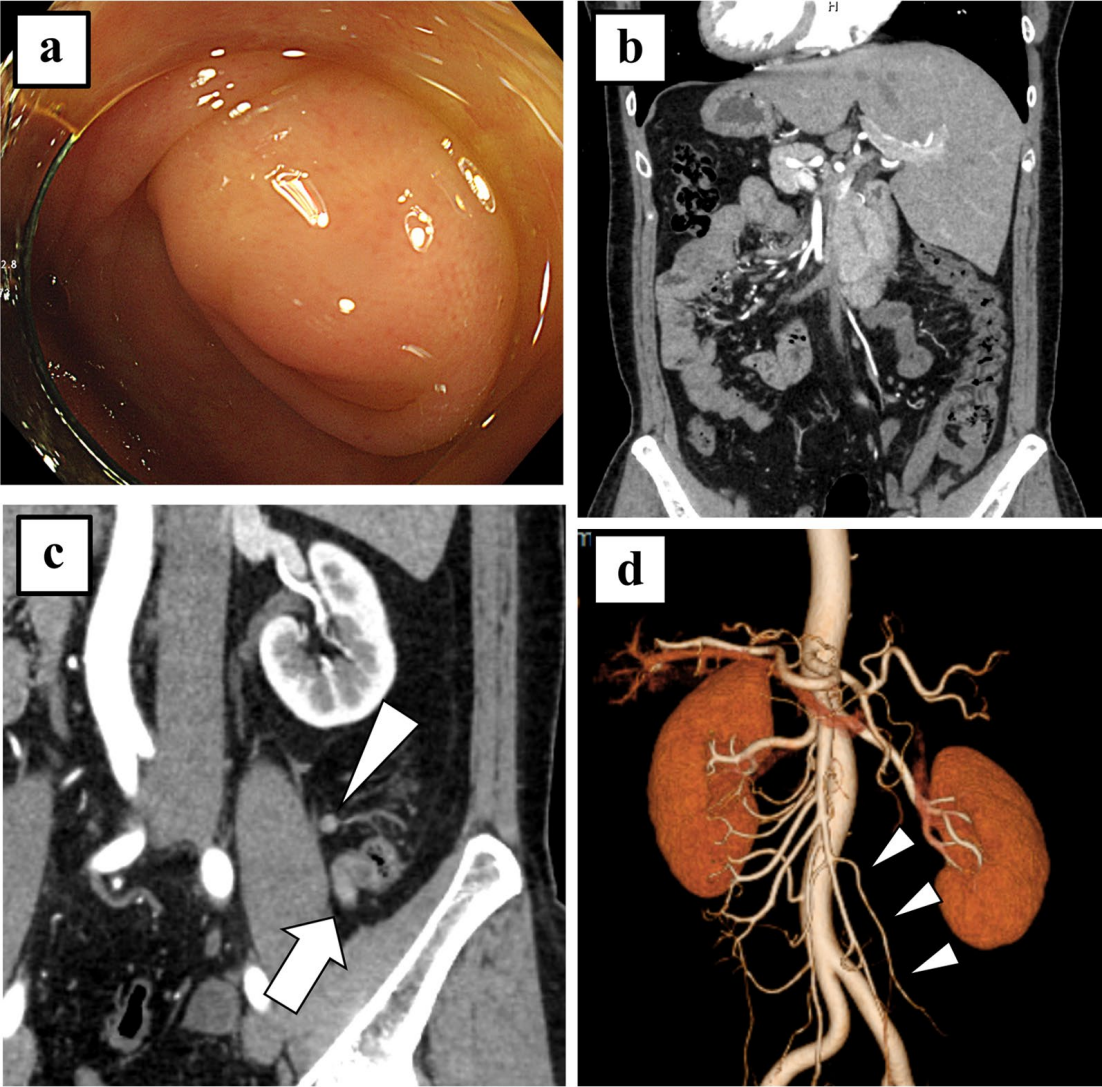
Preoperative examinations. **a** Colonoscopy showing a bulge at the appendiceal orifice. **b** Abdominal contrast-enhanced computed tomography (CT) showing complete “mirror-images” of the visceral organs; situs inversus totalis. **c** CT also showing appendiceal wall thickening, a cystic tumor with contrast effect (arrow), and an enlarged lymph node close to the tumor (arrowhead). **d** 3D-CT showing no vascular anomalies, except for completely inverted vessels. The arrow heads indicate the ileocolic artery

A 41-year-old man with SIT since early childhood was referred to our hospital because of high serum carcinoembryonic antigen levels (6.0 µg/L). The patient had no surgical history. His body mass index was 24.3 kg/m^2^. Physical examination results were normal. All laboratory data were within the normal range, except for the tumor markers. Colonoscopy revealed a bulge at the orifice of the appendix, but pathological examination did not reveal any malignancy (Fig. 1a). Abdominal contrast-enhanced computed tomography (CT) showed complete “mirror-images” of the visceral organs (Fig. 1b). CT also showed appendiceal wall thickening, a cystic tumor with contrast effect, and an enlarged lymph node close to the tumor (Fig. 1c). CT and magnetic resonance imaging showed no solid component in the cystic tumor that would strongly suggest mucinous adenocarcinoma. The preoperative diagnosis was an appendiceal mucocele, which was considered a possible tumor such as low-grade appendiceal mucinous neoplasm (LAMN). We planned a laparoscopic-assisted ileocecal resection with D2 lymph-node dissection since the tumor was located at the root of the appendix with an enlarged lymph node. Preoperatively, we evaluated anatomical variations using 3D-CT, and no vascular anomalies except for completely inverted vessels were observed (Fig. 1d). In addition, we watched horizontally flipped videos of patients with normal anatomy undergoing similar operations to simulate mirror images and symmetrical procedures.

**Fig. 2:**
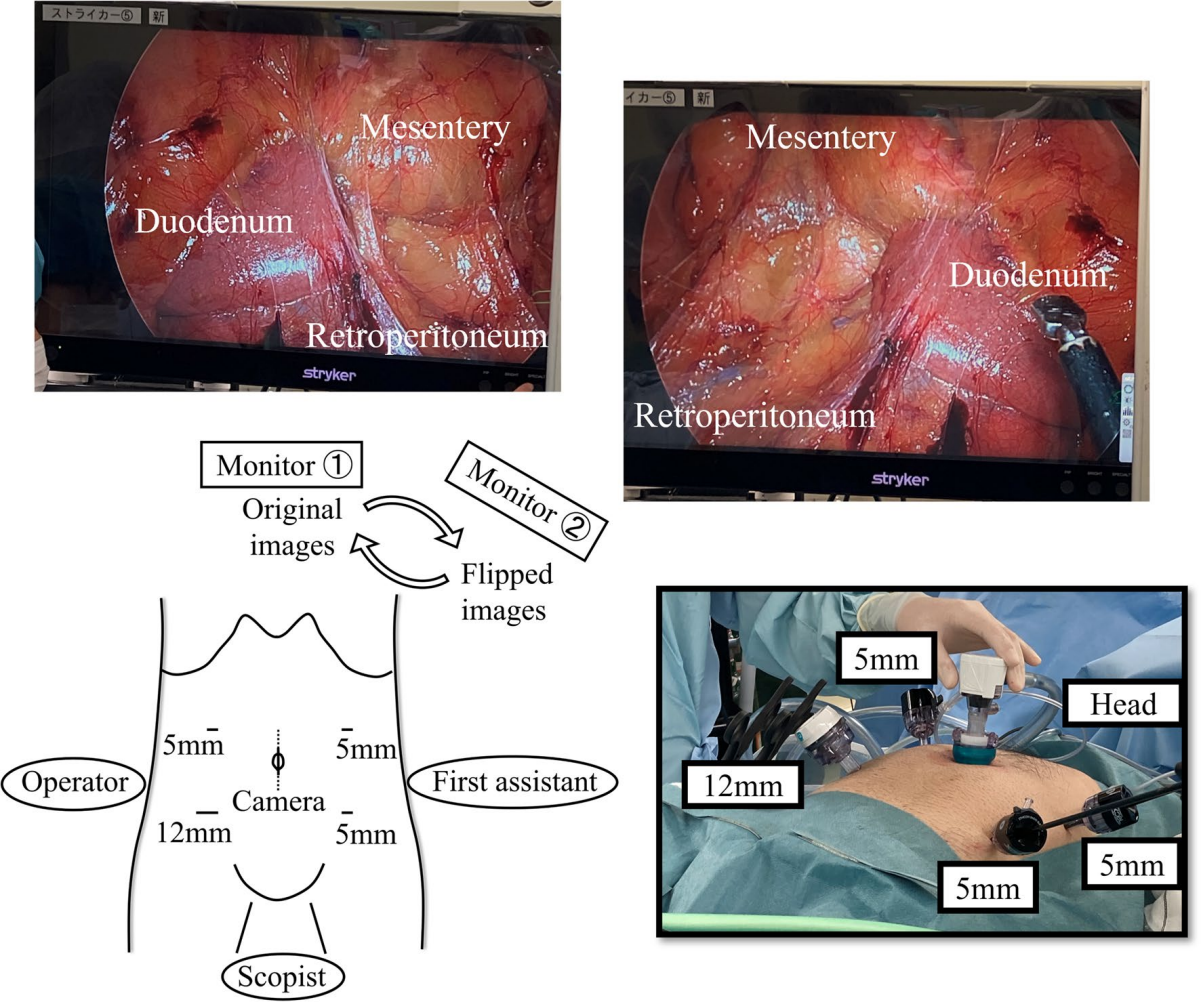
Location of surgeons, trocars, and monitors. The surgeons’ standing positions and trocar placements were reversed from normal. Two monitors were placed at the patient’s head. One monitor showing original images, while the other showing flipped images that looked the same as the normal anatomy. The images displayed on the monitors were exchanged according to the surgical situation

**Fig. 3:**
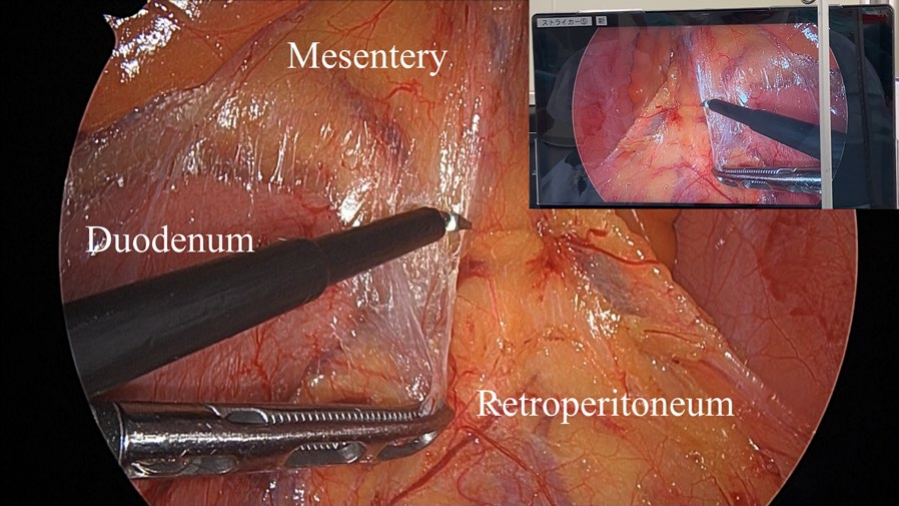
The situation of the surgery. The range of the mobilized region and important anatomical structures were checked by watching the flipped monitor, and the subtle confusion and misrecognition regarding the unfamiliar anatomy was corrected as needed

Under general anesthesia, the patient was placed in lithotomy position. In contrast to normal surgery, the operator stood on the patient’s right side, the first assistant on the left side, and the scopist between the legs (Fig. 2). A laparoscope was inserted through the umbilical trocar, and the other four trocars were placed opposite to their usual placement as shown in Fig. 2. Additionally, a 12 mm trocar was placed in the operator’s right hand, and two monitors were placed at the patient’s head. One monitor showed original images, and the other showed horizontally flipped images that looked the same as the normal anatomy (Fig. 2). The central monitor 1 displayed the original images for the surgeons to see them easily, because it is dangerous and difficult to move the forceps while looking at flipped images due to paradoxical movement of the instruments. Moreover, the images displayed on the monitors were exchanged according to the surgical situation. As needed, the operation was momentarily paused to check for the range of mobilized regions and to visualize important anatomical structures by watching the monitor that showed flipped images (Fig. 3).

**Fig. 4:**
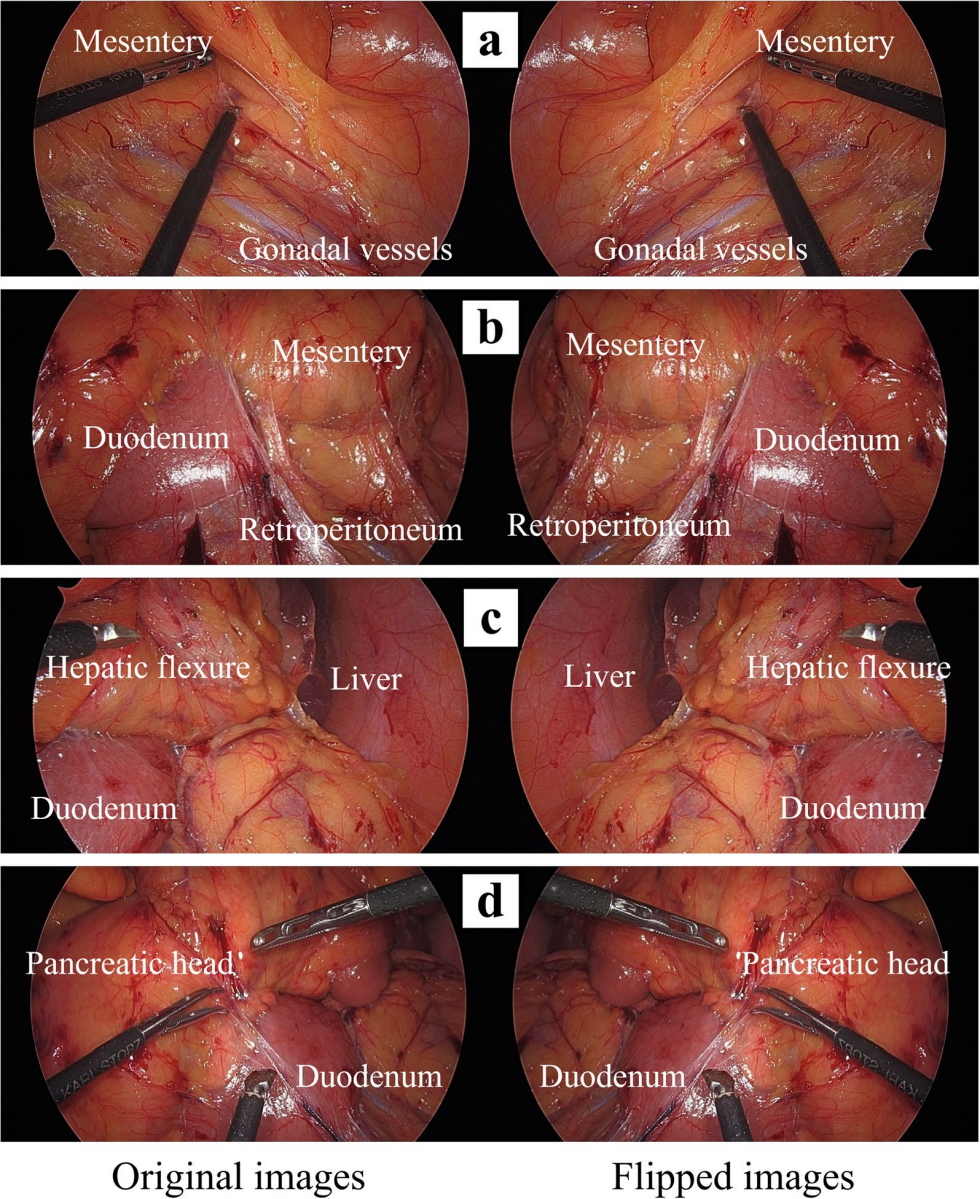
Operative findings with horizontally flipped images. **a** The mesentery of the ileocecum was dissected from the retroperitoneal tissue, paying close attention to the gonadal vessels. **b** The transverse portion of the duodenum was identified, and dissection was performed along the descending portion of the duodenum. **c** The hepatic flexure was mobilized. **d** Additional dissection around the duodenum and pancreatic head was performed

Laparoscopy and intraperitoneal observation revealed transposition of the visceral organs, such as the liver, gallbladder, stomach, and colon. The ileocecal resection procedure was performed using the retroperitoneal approach as usual. The small intestine was moved cranially to secure the surgical field, and we initiated ileocecal mobilization. We dissected the mesentery from the retroperitoneal tissue with a focus on the gonadal vessels (Fig. 4a) and identified the transverse portion of the duodenum. Next, while dissecting along the descending portion of the duodenum (Fig. 4b), we dissected the lateral attachment of the colon to the left abdominal wall toward the cranial side and mobilized the hepatic flexure (Fig. 4c). Finally, we performed additional dissection around the duodenum and pancreatic head, completing the mobilization (Fig. 4d). Since D3 lymph-node dissection was not necessary, we divided the ileocolic vessels near its root without lymph node dissection around the superior mesenteric vein (SMV) and performed resection and reconstruction of the colon extracorporeally. In total, the operative time was 119 min, and the patient’s postoperative course was uneventful. Postoperative pathological examination revealed lymphoid follicles in the intestinal epithelium of the appendiceal orifice and inflamed appendiceal mucosa with neutrophils and eosinophils. No tumor cells suggestive of LAMN or malignancy were observed.

Regarding laparoscopic surgical procedure for SIT, it was unclear how far the mobilization proceeded due to the mirror image; however, during the procedure, we periodically examined the mobilization progression by momentarily pausing the operation to watch the monitor showing flipped images. Additionally, we noted the following differences between surgery in SIT and surgery in patients with normal anatomy: (1) operability involving large movements such as moving the small intestine and securing the surgical field (2) recognition of anatomies such as orientation of the gonadal vessels and duodenum, positional relationship between the hepatic flexure and duodenum, and the hepatic flexure in the upper left abdomen being closer than expected as compared to the splenic flexure in normal anatomy. In such situations, we were able to appropriately address any confusion and misrecognition by checking the flipped monitor (Figs. 3, 4a–c). Additionally, this procedural method allowed for safe operation on important organs, such as the pancreatic head (Fig. 4d). To enable the readers to understand the procedure, a video of the surgery with flipped images has been attached as a Additional file 1: Video S1.

## Discussion

In over 50% of the cases, the initial presentation of appendiceal tumor is acute appendicitis, and the diagnosis usually occurs after appendectomy. Preoperative diagnosis of an appendiceal tumor is difficult because it is infrequently suspected before surgery for appendicitis, and if suspected, performing a colonoscopy and a biopsy is challenging [4]. SIT has been found to further complicate the diagnostic process and delay the therapeutic management of appendiceal disease due to its rare location in the left lower quadrant [5, 6]. Although most people with SIT are asymptomatic, early diagnosis of anatomical anomalies has become more frequent due to the increasingly common use of fetal morphology ultrasound and radiological examinations [6]. SIT is an autosomal recessive congenital disease that is characterized by developmental defects during embryogenesis. Although SIT does not have any pathophysiologic significance and is not a premalignant condition, it has been suggested that patients with SIT may have a higher risk of cancer [7, 8]. In our case, the patient had no history of appendicitis and was known to have SIT since early childhood. Malignancy was suspected because of high levels of tumor markers, and an appendiceal tumor was found by CT and colonoscopy. The patient ultimately underwent surgery because an appendiceal tumor such as LAMN could not be ruled out. Ileocecal resection with D2 lymph-node dissection was performed, considering that D3 lymph-node dissection may be over-invasive with surgical risks. Appendiceal mucinous adenocarcinoma has previously been found to coexist with SIT; however, no neoplastic lesion was observed in our case [5].

Previous reports have also found that SIT often co-exists with various congenital anomalies of the intraabdominal viscera as well as cardiac and renal anomalies, such as syndromes of splenic anomalies (asplenia and polysplenia), anomalies of the hepatobiliary system, intestinal malrotation, agenesis of the dorsal pancreas, preduodenal portal vein, and various vascular anomalies [9–15]. The frequency of cardiovascular anomalies in patients with SIT is reportedly 10 times greater than that in patients with normal anatomy [15]. Therefore, preoperative evaluations using 3D-CT and horizontally flipped videos are useful for understanding the anatomy of vessels and organs and for planning surgical procedures [2, 3, 16]. Due to the mirror-image anatomy, it can be difficult to operate with the right hand, which is the dominant hand in most people. As a result, it is sometimes necessary to use the non-dominant hand or perform a procedure that differs from the usual approach [15, 17]. Case-by-case modifications of port placement and surgeons’ standing positions are also important to improve operability, which are often reversed from normal [2, 3, 16, 18].

Preoperative preparation and planning during surgery are important for patients with SIT, and it is critical to remain aware of the mirror-image anatomy and to perform the operation carefully [13]. When performing fine dissection, we felt less discomfort when viewing mirror images because of the magnified-view effect; however, the field of vision tends to narrow during laparoscopic surgery. Furthermore, important anatomical structures appear from and orient toward unusual directions due to the SIT. In our case study, dissection was sometimes performed in a direction different from what was expected due to the unfamiliar operability. Various factors, including unexpected malformations can cause confusion and anatomical misidentification during operation, which can lead to serious complications particularly in critical situations. For example, right-sided colon surgery includes operations around the duodenum, pancreatic head, and the SMV. In addition, operability deteriorates in operations involving large movements (such as moving the small intestine and securing the surgical field) due to the mirror-image anatomy, and there is a risk of damaging the bowel or mesentery due to improper traction. Even for experts, it is difficult to recognize and correct subtle differences during surgery in unfamiliar situations.

**Fig. 5:**
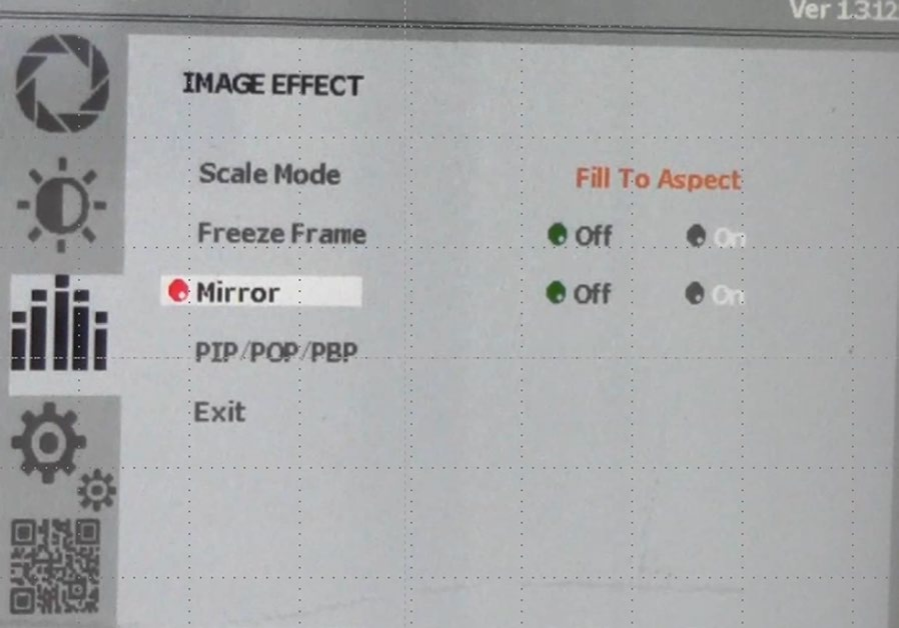
The setting screen of an intraoperative flipped monitor. To turn on the monitor, the mirror button on the image effect page must be pressed

An intraoperative flipped monitor is easily set up by turning on the mirror button on the image effect page of the monitor setting screen (Fig. 5). Therefore, the images displayed on the monitors can easily be switched depending on the surgical situation without slowing down the operation. Checking the flipped monitor as needed to observe the normal anatomy and correct any misrecognition reduces the risk of complications due to anatomical misidentification. Kigasawa et al. reported the usefulness of this technique during laparoscopy-assisted distal gastrectomy [19]. An intraoperative flipped monitor is especially useful when checking the secureness of the surgical field, range of mobilized regions, progression of dissection, appearance of new anatomical structures, presence of vascular and intestinal malformations, and operation of critical organs. It is also useful before dividing blood vessels and reattaining orientation after it is lost. Moreover, other members of staff present in the room, but not essentially operating, carefully observed the monitor showing flipped images and provided appropriate suggestions to the operating surgeons. The aforementioned practices are especially useful in more difficult surgeries and is advantageous for use in laparoscopic surgery for SIT. Since this flipping technique can be applied to any procedure that utilizes monitors, an intraoperative flipped monitor may also be useful for other endoscopic procedures.

## Conclusions

We report a case of laparoscopic-assisted ileocecal resection of an appendiceal tumor in a patient with SIT using an intraoperative flipped monitor. The intraoperative flipped monitor was easy to set up and was helpful in reducing the risk of anatomical misidentification during surgery.

### Supplementary Information

**Additional file 1: Video S1.** Usefulness of an intraoperative flipped monitor in laparoscopic surgery with situs inversus totalis: a video of laparoscopic-assisted ileocecal resection with flipped images.

## Data Availability

Not applicable.

## Abbreviations

CT: Computed tomography
LAMN: Low-grade appendiceal mucinous neoplasm
SIT: Situs inversus totalis
SMV: Superior mesenteric vein
